# Circulating autotaxin levels in healthy teenagers: Data from the Vitados cohort

**DOI:** 10.3389/fped.2023.1094705

**Published:** 2023-02-13

**Authors:** Marie-Noëlle Méaux, Maitena Regnier, Aurélie Portefaix, Olivier Borel, Candide Alioli, Olivier Peyruchaud, Mélanie Legrand, Justine Bacchetta

**Affiliations:** ^1^ INSERM, UMR 1033, Lyon, France; ^2^ Centre de Référence des Maladies Rares du Calcium et du Phosphate, filière OSCAR, Lyon, France; ^3^ Service de Néphrologie, Rhumatologie et Dermatologie Pédiatriques, Hôpital Femme Mère Enfant, Hospices Civils de Lyon, Bron, France; ^4^ Centre d’Investigation Clinique, CIC 1407, Hospices Civils de Lyon, Bron, France; ^5^ Faculté de Médecine Lyon Est, Université Claude Bernard Lyon 1, Lyon, France; ^6^ Service de Rhumatologie, Hôpital Edouard Herriot, Hospices Civils de Lyon, Lyon, France

**Keywords:** autotaxin (ATX), teenagers, inflammation, cardiovascular risk, puberty age

## Abstract

Autotaxin (ATX) is a secreted enzyme with a lysophospholipase D activity, mainly secreted by adipocytes and widely expressed. Its major function is to convert lysophosphatidylcholine (LPC) into lysophosphatidic acid (LPA), an essential bioactive lipid involved in multiple cell processes. The ATX-LPA axis is increasingly studied because of its involvement in numerous pathological conditions, more specifically in inflammatory or neoplastic diseases, and in obesity. Circulating ATX levels gradually increase with the stage of some pathologies, such as liver fibrosis, thus making them a potentially interesting non-invasive marker for fibrosis estimation. Normal circulating levels of ATX have been established in healthy adults, but no data exist at the pediatric age. The aim of our study is to describe the physiological concentrations of circulating ATX levels in healthy teenagers through a secondary analysis of the VITADOS cohort. Our study included 38 teenagers of Caucasian origin (12 males, 26 females). Their median age was 13 years for males and 14 years for females, ranging from Tanner 1 to 5. BMI was at the 25th percentile for males and 54th percentile for females, and median blood pressure was normal. ATX median levels were 1,049 (450–2201) ng/ml. There was no difference in ATX levels between sexes in teenagers, which was in contrast to the male and female differences described in the adult population. ATX levels significantly decreased with age and pubertal status, reaching adult levels at the end of puberty. Our study also suggested positive correlations between ATX levels and blood pressure (BP), lipid metabolism, and bone biomarkers. However, except for LDL cholesterol, these factors were also significantly correlated with age, which might be a confounding factor. Still, a correlation between ATX and diastolic BP was described in obese adult patients. No correlation was found between ATX levels and inflammatory marker C-reactive protein (CRP), Body Mass Index (BMI), and biomarkers of phosphate/calcium metabolism. In conclusion, our study is the first to describe the decline in ATX levels with puberty and the physiological concentrations of ATX levels in healthy teenagers. It will be of utmost importance when performing clinical studies in children with chronic diseases to keep these kinetics in mind, as circulating ATX might become a non-invasive prognostic biomarker in pediatric chronic diseases.

## Introduction

Autotaxin (ATX), also known as ENPP2 (ecto-nucleotide pyrophosphatase phosphodiesterase family member 2), is a secreted enzyme with a lysophospholipase D activity (1–4). ATX is the only secreted member of the ENPP family (5), synthesized by different cell types and mainly by adipocytes (6). ATX is found in numerous biological fluids and tissues (7), including blood, with a short circulating half-life due to a quick clearance by the liver (8). Its pleiotropic expression likely reflects its role in multiple physiological processes.

ATX converts lysophosphatidylcholine (LPC) into lysophosphatidic acid (LPA) (1), the smallest bioactive glycerophopholipid (9). LPA binds to at least six protein G-coupled receptors, LPA 1–6 (10), that are differentially expressed in tissues with distinct and overlapping biological responses, thus explaining the large range of cellular processes involving LPA (6). LPA is one of the smallest glycerophospholipids of the organism (5) and a structural component of cellular membranes (11). It is also a major bioactive lipid acting as a signaling molecule involved in multiple cell processes including survival, migration, and proliferation, among almost all cell types (9).

LPA and ATX are crucial in different systems, notably reproduction (ovarian function, embryo implantation, maintenance of pregnancy) (10, 12, 13), development of the central nervous system (14), immunity (15, 16), inflammation (17), and lipid metabolism (18). A ubiquitous deletion of ATX leads to early embryonic lethality in mice (19), notably because of defective vasculogenesis (20, 21) and central nervous system development (14).

With regard to inflammation, ATX transcription is enhanced by pro-inflammatory cytokines such as tumor necrosis factor alpha (TNFα) in hepatocytes through the nuclear factor kappa beta (NFkB) signaling pathway (10, 22) and IL-6 in adipocytes (23). Alternatively, ATX will induce the production of pro-inflammatory cytokines, thus maintaining inflammation (17). With regard to lipid metabolism, the adipose tissue is one of the main sources of ATX (6, 24), and ATX production by adipocytes is increased in the presence of pro-inflammatory cytokines (23). ATX increases pre-adipocyte proliferation (6), but its role in pre-adipocyte differentiation is more controversial, with a probably inhibitory effect (18). Through this pathway, the ATX–LPA axis contributes to obesity and insulin resistance (18).

Thus, the ATX–LPA axis is involved in various physiological processes, but it is also linked to several pathological conditions, especially inflammatory and neoplastic diseases (17, 25). The assessment of circulating ATX levels might therefore be interesting for clinical applications.

Studies have shown that measurement of serum ATX antigen level is a reliable indicator of ATX activity in human serum samples (26, 27) and thus can be used for clinical investigation. Circulating levels of ATX have been reported in healthy adults: ATX levels are significantly greater in women (625–1,323 ng/ml) than in men (438–914 ng/ml) (13, 26, 28). ATX levels are also negatively correlated with age in adult men, but it is not correlated with age in women (26). Moreover, the serum phospholipase D/ATX activity gradually increases during pregnancy (4, 29) and so do ATX levels: serum ATX antigen is correlated to gestational week (13), increasing from 1,961 ± 450 ng/ml at the first semester to 5150 ± 2,143 ng/ml at the third trimester of pregnancy, with a further decrease immediately after delivery. ATX levels have also been described in pathological conditions such as inflammatory or hepatic diseases. For example, it gradually increases with fibrosis stage in chronic hepatitis C, and even correlates in biliary atresia with histological fibrosis grades, increasing from 1,080 ng/ml at F0 grade to 2,500 ng/ml at F4 grade (27, 28, 30). ATX serum levels are also influenced by the metabolic status of the individuals and correlate with insulin resistance in obese patients and with their body mass index (BMI) (31). A recent study even revealed a rapid and lasting decrease in ATX levels after bariatric surgery in obese patients, reinforcing the connection between ATX and lipid metabolism (32).

However, no data have been published on the pediatric age. Thus, the aim of our work is to describe the physiological concentrations of circulating ATX levels in healthy teenagers through a secondary analysis of the VITADOS cohort (33) and to screen for potential correlations with other circulating biomarkers as a basis for further research on the pathological role of ATX in chronic diseases affecting teenagers.

## Subjects and methods

### Population

The initial aim of the Vitados cohort (NCT01832623) was to assess native vitamin D [25-(OH)-D] status in a general French population of healthy Caucasian teenagers in association with their bone and cardiovascular status; it allowed to describe normal values for the main phosphate/calcium and bone biomarkers in the total cohort of 100 included healthy teenagers depending on sex and puberty (30). Exclusion criteria were the following: walking disability, past or ongoing treatment by growth hormone (rhGH) therapy, past intake of oral corticosteroids (for more than 3 months), ongoing treatment with corticosteroids or calcineurin inhibitors, chronic disease with a likely effect on growth (and notably chronic parenteral nutrition, chronic inflammatory disease, systemic disease, chronic renal insufficiency, diabetes mellitus), acute ongoing severe disease (and notably infection or cancer), pregnancy, and an intake of acetylsalicylic acid or anti-inflammatory drugs within the last 3 weeks. Here, we were able to work on the remaining sera of 38 subjects from this cohort.

Demographic, physical, and biochemical data were recorded. Height and weight were presented as the standard deviation score (SDS) for age and sex, and BMI was presented as the percentile for age and sex. The Tanner stage was assessed by an experienced physician. Systolic and diastolic blood pressure (SBP and DBP) were expressed in terms of percentile according to age, sex, and height (34).

### Blood samples

Morning fasting samples were obtained. Because of the peculiar evolution of phosphate levels across the pediatric age, phosphate levels were expressed as the standard deviation score (SDS) for age (35). Renal function was estimated using the 2009 Schwartz formula to calculate an estimated glomerular filtration rate (eGFR) (36). All assays used for standard biochemical assessments were ones that were previously described (33).

### ATX measurements with ELISA assays

To measure ATX levels in serum, we used ATX sandwich ELISA kits manufactured by Echelon Biosciences (K-5600). Samples were placed on an ATX detection plate (K-5601), and an anti-ATX antibody (K-5603) was used and revealed by a secondary detector (K-SEC7). The revelation was made by using absorbance assays. The normal human serum level of ATX described in this kit is 589–1,135 ng/ml. A volume of 10 µl was used and every sample was assessed in duplicate.

### Statistical analysis

The results were presented as median (min−max). Non-parametric tests were performed: Mann–Whitney tests, Kruskall–Wallis tests for multiple comparisons followed by Dunn's tests, and Spearman tests for correlation. A *p*-value below 0.05 was considered statistically significant. Statistical analyses and figures were performed with Graphpad Prism 8.

### Ethical committee

The VITADOS study was approved by the Comité de Protection des Personnes Lyon Sud Est II; all patients and parents (or legal guardians) gave their consent after written information.

## Results

### Description of the cohort and results on ATX levels

The demographic and biological characteristics of the subgroup of the VITADOS cohort studied here are given in Table 1.

**Table 1:** Demographic and biochemical characteristics of the subjects used for the secondary analysis of the VITADOS cohort.

	Males (*n* = 12)	Females (*n* = 26)
Age (years)	13.3 (10.2–17.8)	14.2 (10.5–17.8)
Tanner stage		
Tanner 1	3	1
Tanner 2	3	4
Tanner 3	2	6
Tanner 4	2	7
Tanner 5	2	8
Height (cm)	161 (136–187)	158 (143–180)
Height (SD)	0.9 (−2.4–2.7)	0.7 (−1.0–4.0)
Weight (kg)	55 (26–77)	47 (32–67)
Weight (SD)	0.2 (−1.7–5.4)	0.8 (−1.0–2.4)
BMI (percentile)	25 (3–99)	54 (20–97)
SBP (percentile)	30 (5–88)	29 (0–94)
DBP (percentile)	34 (9–93)	32 (2–92)
Creatinine (µmol/L)	55 (43–94)	54 (40–80)
eGFR (ml/min/1.73 m^2^)	107 (71–129)	107 (74–141)
Calcium (mmol/L)	2.42 (2.24–2.57)	2.37 (2.20–2.53)
Phosphate (mmol/L)	1.40 (1.08–1.60)	1.38 (0.93–1.61)
Phosphate SDS	−0.6 (−1.6–0.4)	−0.6 (−2.3–0.5)
PTH (ng/L)	17 (10–24)	17 (11–29)
ALP (UI/L)	229 (84–524)	176 (54–305)
25OHD (nmol/L)*	55 (30–66)	65 (48–129)
1,25(OH)_2_D_3_ (pmol/L)	123 (98–173)	142 (96–206)
FGF23 (UI/L)	62 (43–98)	74 (58–106)
ALP (UI/L)	229 (84–524)	176 (55–305)
BAP (UI/L)	62 (17–194)	57 (11–110)
CTX (µmol/L)*	1,746 (1442–2410)	1,414 (652–2260)
OCN (µg/L)	71 (40–160)	64 (27–238)
Total Ch (mmol/L)	4.2 (3.3–5.1)	4.3 (2.3–5.7)
LDL Ch (mmol/L)	2.4 (1.7–3.2)	2.5 (0.8–4.0)
HDL Ch (mmol/L)	1.2 (0.8–2.1)	1.5 (0.9–2.3)
TG (mmol/L)	0.7 (0.3–1.9)	0.6 (0.3–1.4)
ATX (ng/ml)	1,109 (521–1455)	977 (450–2201)

Results are expressed as median (min–max).

cm, centimeters; kgs, kilograms; SD, standard deviation; BMI, body mass index; SBP, systolic blood pressure; DBP, diastolic blood pressure; SDS, standard deviation score; ALP, alkaline phosphatases; 25OHD, 25-hydroxy-vitamin D; 1,25(OH)_2_D_3_, 1,25-dihydroxy-vitamin D; FGF23, fibroblast growth factor 23; ALP, alkaline phosphatases; BAP, bone ALP; CTX, C-terminal fraction of type 1 collagen; OCN, osteocalcin; Ch, cholesterol; LDL Ch, low-density lipoprotein cholesterol; HDL Ch, high-density lipoprotein cholesterol; TG, triglycérides; ATX, autotaxin; eGFR, estimated glomerular filtration rate.

Comparison was made by using the Mann–Whitney test. *p < 0.05.

**Figure 1:**
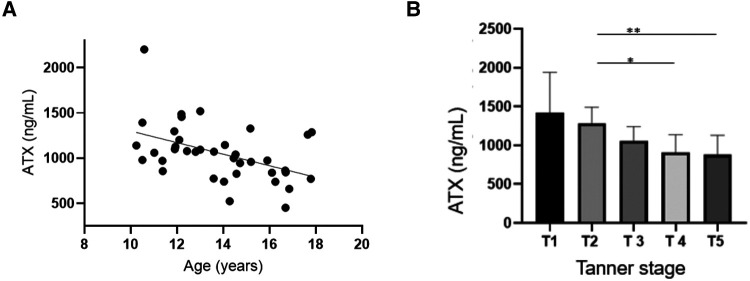
Autotaxin is negatively associated with age and pubertal status. (**A**) Correlation tests were conducted between ATX circulating levels (ng/ml) and age Spearman correlation test: *r* = −0.47, IC95% = −0.69−(−)0.16, *p* = 0.0034 **. (**B**) Correlation tests were performed between ATX circulating levels (ng/ml) and the Tanner stage. Kruskal–Wallis test: *p* = 0.0039. *p* (T2 vs. T5) = 0.025 *, *p* (T2 vs. T4) = 0.049 *.

The ATX median levels were 1,049 (450–2,201) ng/ml. There was no difference in the ATX levels in terms of sex, but these levels significantly decreased with age and pubertal status, as illustrated in Figure 1, Table 2. There was no relation either with regard to normalized height or body weight or with regard to BMI percentile.

**Table 2:** Normal values of ATX according to sex and pubertal status.

ATX concentration (ng/ml)	Tanner stage		
	1–2 (*n* = 6M 5F)	3 (*n* = 2M 6F)	4 −5 (*n* = 4M 15F)
Male	1,132	1,119	714
Median (min−max)	(1,071–1,455)	(1,093–1,145)	(521–1,259)
Female	1,485	1,010	862*
Median (min−max)	(1,058–2,201)	(739–1,391)	(450–1,327)

Results are expressed as median (minimum – maximum) and as mean (standard deviation).

ATX, autotaxin; M, male; F = female. For each component, the number of available data is precised (*n*).

*Statistical difference compared with Tanner stage 1–2, *p* = 0.0045.

### ATX and cardiovascular markers

**Figure 2:**
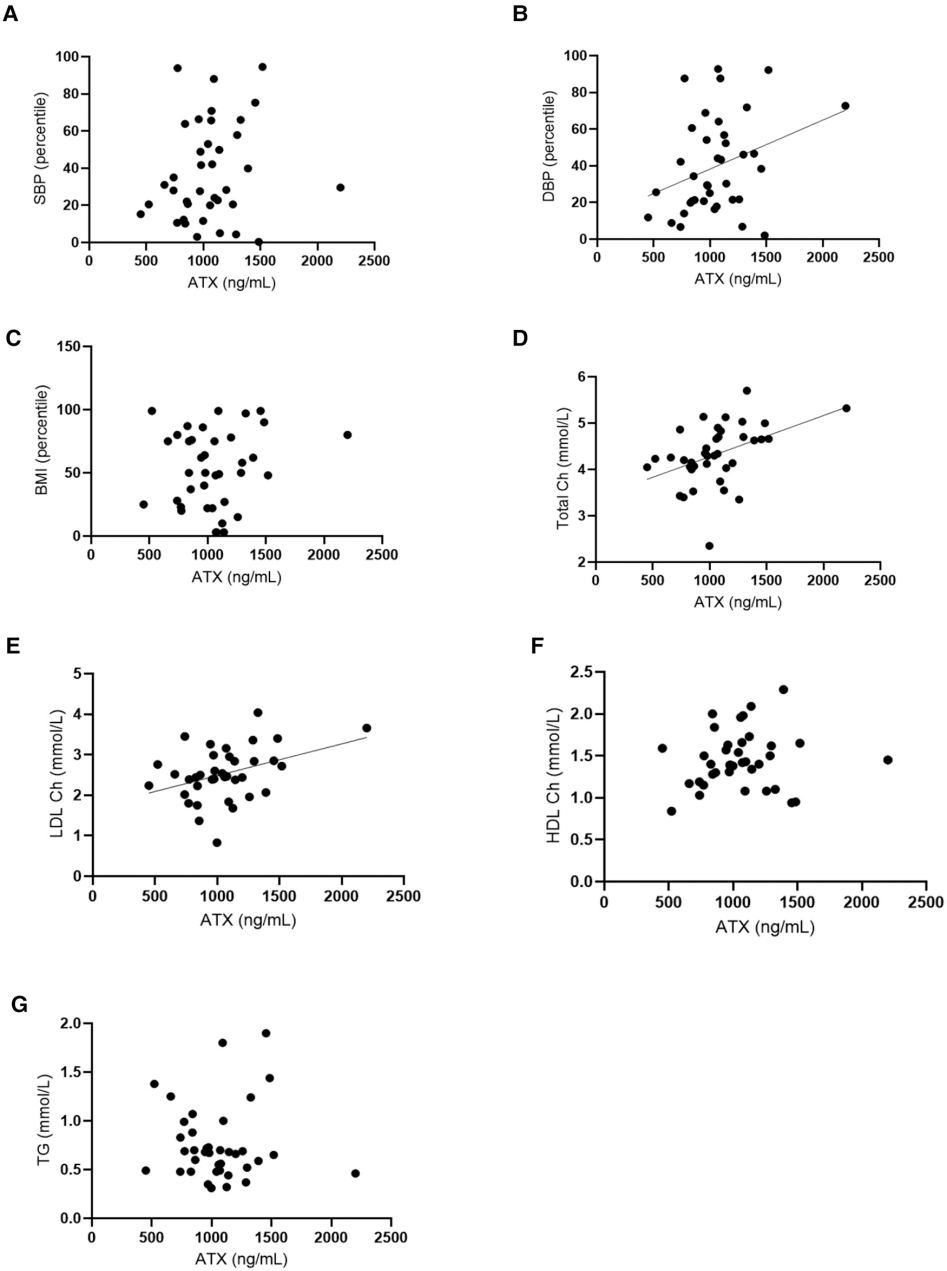
Autotaxin and age are positively correlated with cardiovascular risk and fat metabolism markers. (**A**) Correlation tests were conducted between ATX circulating levels (ng/ml) and systolic blood pressure (SBP, percentile). Spearman correlation test: *r* = 0.34, IC95% = 0.01–0.61, *p* = 0.04. (**B**) Correlation tests were performed between ATX circulating levels (ng/ml) and diastolic blood pressure (DBP, percentile). Spearman correlation test: *r* = 0.34, IC95% = 0.01–0.61, *p* = 0.04. (**C**) Correlation tests were done between ATX circulating levels (ng/ml) and body mass index (percentile). Spearman correlation test: non-significant. (**D**) Correlation tests were carried out between ATX circulating levels (ng/ml) and total cholesterol (Total Ch) (mmol/L). Spearman correlation test: *r* = 0.46, IC95% = 0.14–0.68, *p* = 0.0046**. (**E**) Correlation tests were done between ATX circulating levels (ng/ml) and LDL cholesterol (LDL Ch) (mmol/L). Spearman correlation test: *r* = 0.33, IC95% = 0.01–0.59, *p* = 0.0433*. (**F**) Correlation tests were performed between ATX circulating levels (ng/ml) and HDL cholesterol (HDL Ch) (mmol/L). Spearman correlation test: non-significant. (**G**) Correlation tests were done between ATX circulating levels (ng/ml) and triglyceride levels (TG) (mmol/L). Spearman correlation test: non-significant. Age is correlated with these for blood factors and the Spearman correlation tests: Age and SBP: non-significant. Age and DBP: *r* = −0.4369, IC95% = −0.67 – (−)0.13, *p* = 0.0061. Age and per BMI: *r* = 0.0168, IC95% = −0.31–0.34, *p* = 0.9204 non-significant. Age and total Ch: *r* = −0.3670, IC95% = −0.62 – (−)0.04, *p* = 0.0234. Age and LDL Ch: *r* = −0.2200, IC95% = −0.61–0.12, *p* = 0.1845 non-significant. Age and HDL Ch: *r* = −0.3649, IC95% = −0.62 – (−)0.014, *p* = 0.0243. Age and TG: *r* = 0.2168, IC95% = −0.12–0.51, *p* = 0.1911 non-significant.

A positive correlation was found between ATX levels and diastolic blood pressure (*p* = 0.003), but later, these also correlated with age. ATX also positively correlated with circulating total and LDL cholesterol (*r* = 0.46, *p* = 0.005 and *r* = 0.33, *p* = 0.043, respectively). Age was negatively correlated with total cholesterol but not with LDL cholesterol. These results are shown in Figure 2.

### ATX and phosphate/calcium and bone biomarker metabolism

**Figure 3:**
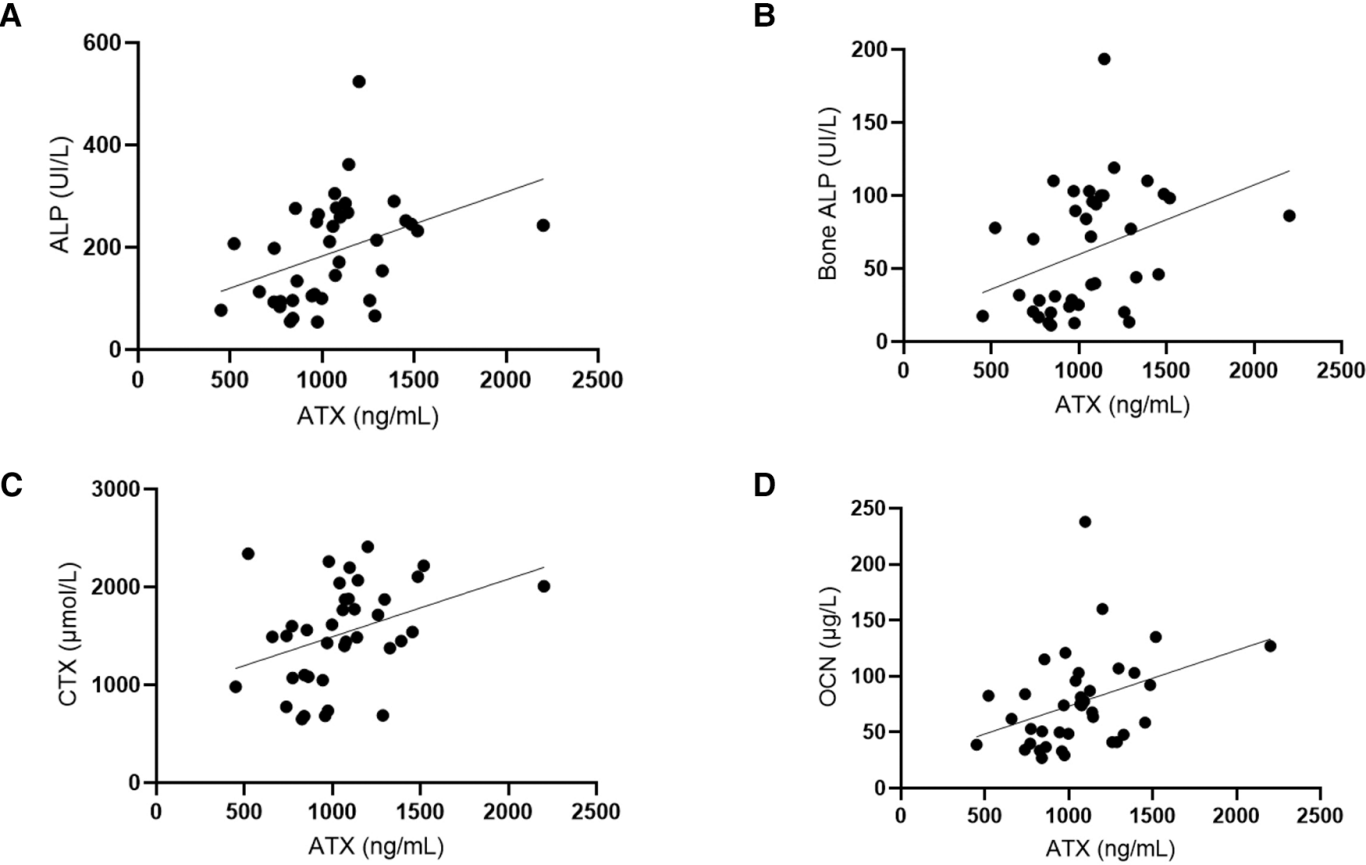
Autotaxin is positively correlated with bone turnover markers. (**A**) Correlation tests were done between ATX circulating levels (ng/ml) and ALP (UI/L, alkaline phosphatases). Spearman correlation test: *r* = 0.051, IC95% = 0.22–0.72, *p* = 0.0011**. (**B**) Correlation tests were performed between ATX circulating levels (ng/ml) and bone ALP (UI/L, bone alkaline phosphatases). Spearman correlation test: *r* = 0.47, IC95% = 0.17–0.69, *p* = 0.0029**. (**C**) Correlation tests were done between ATX circulating levels (ng/ml) and CTX (µmol/L, C terminal fraction of type 1 collagen). Spearman correlation test: *r* = 0.42, IC95% = 0.11–0.66, *p* = 0.0086**. (**D**) Correlation tests were done between ATX circulating levels (ng/ml) and OCN (µg/L, osteocalcine). Spearman correlation test: *r* = 0.45, IC95% = 0.15–0.68, *p* = 0.0043**. Age was also correlated with these biomarkers and the Spearman correlation tests: Age and ALP: *r* = −0.7944, IC95% = −0.89 – (−)0.63, *p* < 0.0001. Age and BAP: *r* = −0.8111, IC95% = −0.90 – (−)0.66, *p* < 0.0001. Age and CTX: *r* = −0.4945, IC95% = −0.71 – (−)0.20, *p* = 0.0016. Age and OCN: *r* = −0.7694, IC95% = −0.88 – (−)0.59, *p* < 0.0001.

There was no correlation between ATX and calcium, phosphate SDS, PTH, 25OHD, 1,25(OH)_2_D_3_, FGF23, or urinary calcium/creatinine ratio. However, ATX levels were statistically associated with the markers of bone turnover: alkaline phosphatase (ALP), bone ALP (BAP), C-terminal fraction of type 1 collagen (CTX), and osteocalcin (OCN). However, all these factors were also significantly correlated with age. These results are shown in Figure 3.

In contrast, no correlation was found between the osteocyte-synthesized sclerostin and ATX or age (*p* = 0.13 and 0.07, respectively).

### ATX and inflammation, kidney function

No correlation was found between the inflammatory marker C-reactive protein (CRP) and with eGFR.

### Multivariate analysis

By using a backward multivariate analysis including age, ALP (as a marker of bone formation), and CTX (as a marker of bone resorption) in the model, nothing remained significantly associated with ATX.

## Discussion

The main objective of this study is to describe normal ATX values in healthy teenagers aged 10–18 years, since data are lacking in this age group. This is of utmost importance for future studies in the field and notably in children with chronic diseases such as chronic inflammatory states or obesity. Indeed, in adults, the deregulation of the ATX–LPA axis has been recently shown in chronic pathological conditions, especially in neoplastic and inflammatory diseases with increased ATX levels (2, 17, 25, 37).

Indeed, ATX was first described in 1992 as an autocrine motility factor in human melanoma (38). Since then, ATX was found overexpressed and implicated in many different cancers such as hepatocellular carcinoma (22), breast cancer (39), glioblastoma (40, 41), Hodgkin lymphoma (42), and non-small-cell lung cancer (43), with a growing evidence that it is directly involved in tumor progression, invasiveness, and dissemination through the production of LPA (2, 5, 44).

However, ATX is also closely linked to inflammation: its levels increase in many inflammatory diseases such as pulmonary fibrosis (45, 46), rheumatoid arthritis (47), or chronic inflammatory bowel diseases (48). In murine models, conditional genetic deletions of ATX or of the LPA-receptor LPA-R1, as well as pharmacological ATX inhibition (45, 47, 49), lead to attenuated inflammation, thus suggesting the contribution of the ATX–LPA axis in the pathogenesis of these diseases.

Because of its synthesis by hepatocytes, ATX is also deregulated in liver diseases: in patients with chronic fibrosis due to hepatitis C, circulating ATX levels increase gradually with fibrosis stage (27, 28). In biliary atresia (BA), ATX levels correlate with histological fibrosis grades (30), increasing from 1,080 ng/ml at F0 grade to 2,500 ng/ml at F4 grade, thus being a potential interesting non-invasive marker for liver fibrosis estimation. In that setting, it is interesting to note that this study was performed in 35 patients at a median age of 10.6 years, and that the results obtained at the F0 stages were similar to those we report here in healthy subjects at a similar age. Another pediatric study was performed directly on liver specimens from infants with BA undergoing Kasai operation (*N* = 20) and compared with samples from infants who underwent liver biopsy for another reason (*N* = 14); interestingly, this study found that mRNA and protein expression of ATX were increased in BA livers (50). High hepatic ATX expression at the time of Kasai operation was associated with liver fibrosis and outcome in BA, suggesting that ATX may serve as a prognostic biomarker in this infantile disease (50). Eventually, ATX levels are associated with cirrhosis grade whatever its cause may be, along with its complications (e.g., hepatic encephalopathy, esophageal varices, and portal hypertensive gastropathy), and it is an independent predictor of overall survival in this population, as demonstrated in a longitudinal cohort of 270 adult patients with liver cirrhosis followed until death, liver transplantation, or last contact (51). Reference values in healthy adult controls were 258 ± 40 ng/ml in this study (51).

The ATX–LPA axis is also linked to lipid metabolism, as previously described, including stimulation of pre-adipocytes proliferation. In mice, adipocyte ATX expression is increased in genetically obese mice in correlation with their insulin resistance state (52). The heterozygous model or adipose-specific knockdown of ATX is associated with attenuated diet-induced obesity and decreased insulin resistance (53), likely through LPAR1 (54). In humans, adipocyte ATX expression is enhanced in subjects with insulin resistance (52), and serum ATX levels correlate with insulin resistance in obese patients (31). Thus, ATX closely links with obesity and insulin resistance both in humans and in mice, with growing evidence of its involvement in the impaired glucose homeostasis of diet-induced obesity. This suggests a potential future therapeutic target of ATX and LPAR1 for the treatment of overweight or diabetes-related metabolic diseases. Interestingly, visceral fat ATX expression was found to be increased in obese female patients (*N* = 27) compared with non-obese patients (*N* = 10) in a previous study (55), which also describes a correlation between ATX and diastolic arterial BP in obese patients, similarly to our present study finding a correlation of ATX levels and diastolic BP in teenagers. However, in our study, this correlation is also found between ATX levels and age, which could then be a confounding factor.

Lastly, the ATX–LPA axis is involved in various other pathological conditions, especially cardiovascular diseases, such as the development of atherosclerosis through the accumulation of LPA in atherosclerotic plaques (56) and the LPA-mediated adventitial mast cell activation leading to vascular inflammation and plaque instability (57).

Its involvement in various pathological processes suggests that ATX might be an interesting prognostic biomarker and also a potential target for the treatment of numerous diseases. Indeed, the inhibition of ATX or LPAR1 is a potential therapeutic strategy in cancer and inflammatory diseases, with promising preclinical results with a good safety profile to date (10, 58).

Here, we found no differences in ATX levels between sexes, contrary to what is observed in adults where ATX levels are significantly higher in women. ATX levels significantly decrease with age and pubertal status, reaching 714 (521–1,259) ng/ml in males and 862 (450–1,327) ng/ml in females at the Tanner stage 4–5, which is consistent with the adult values in the literature previously described. Moreover, as mentioned earlier, a previous study also described decreased ATX levels with age among male adults (26) but not among women. Thus, we can hypothesize a potential interaction between sexual hormones and ATX levels because of the sex difference in adults on the one hand and because of the modification of ATX levels during pregnancy on the other hand. Here, we show that, similarly to other biomarkers [and notably phosphate levels (59)], ATX levels decrease along puberty in healthy teenagers. To our knowledge, this is the first description of a modification of ATX levels with puberty in humans. The fact that ATX is deregulated in some gynecological conditions reinforces the hypothesis of an association between sexual hormones and ATX: indeed, in endometrial carcinomas, ATX mRNA expression is higher in neoplastic cells that are positive for estrogen receptor (ER) than in ER-negative neoplastic cells (60). Moreover, in endometrial cancer, estrogens stimulate ATX expression, and the ATX-LPA axis is involved in estrogen cell proliferation through the MAPK-ERK signaling pathway (60).

This pilot study also suggested positive correlations between ATX levels and blood pressure, lipid metabolism, and bone biomarkers but not between biomarkers of phosphate/calcium metabolism. These results should be confirmed in larger studies, especially because age could be a confounding factor in these bivariate analyses. Still, a correlation between ATX and diastolic BP has been described in obese adult patients (55). Alternatively, the positive correlation between ATX and LDL cholesterol, which seems independent of age, is consistent with the implication of the ATX-LPA axis in lipid metabolism.

This study has several strengths, including a well-phenotyped prospective transversal cohort of healthy pediatric subjects. We measured the biomarkers of bone and phosphate/calcium metabolism using the most recent available assays and were, therefore, able to provide data depending on sex and pubertal status. According to the guidelines from the Clinical and Laboratory Standards Institute (CLSI), 120 healthy subjects per group are required to establish pediatric reference values, with a minimum of 20 subjects per group to validate existing data. As such, we cannot consider that here we provide reference values, especially because we had serum sufficient for only a subset of the VITADOS subjects. Thus, we may have lacked the power to demonstrate differences according to sex.

In conclusion, we are the first to describe the decline in ATX levels with puberty. It will be of utmost importance to keep these kinetics in mind when performing clinical studies in children with chronic diseases, as circulating ATX might become a non-invasive prognostic biomarker in pediatric chronic diseases.

## Data Availability

The raw data supporting the conclusions of this article will be made available by the authors without undue reservation.
